# Phenomenal Causality and Sensory Realism

**DOI:** 10.1177/2041669520927038

**Published:** 2020-06-01

**Authors:** Kristof Meding, Sebastian A. Bruijns, Bernhard Schölkopf, Philipp Berens, Felix A. Wichmann

**Affiliations:** Neural Information Processing Group, Eberhard Karls Universität Tübingen; Empirical Inference Department, Max-Planck-Institute for Intelligent Systems, Tübingen, Germany; Neural Information Processing Group, Eberhard Karls Universität Tübingen; Empirical Inference Department, Max-Planck-Institute for Intelligent Systems, Tübingen, Stuttgart, Germany; Institute for Ophthalmic Research, Eberhard Karls Universität Tübingen; Neural Information Processing Group, Eberhard Karls Universität Tübingen

**Keywords:** causal perception, intuitive physics, cue combination, cue conflict, Albert Michotte

## Abstract

One of the most important tasks for humans is the attribution of causes and effects in all wakes of life. The first systematical study of visual perception of causality—often referred to as *phenomenal causality*—was done by Albert Michotte using his now well-known *launching events* paradigm. Launching events are the seeming collision and seeming transfer of movement between two objects—abstract, featureless stimuli (“objects”) in Michotte’s original experiments. Here, we study the relation between causal ratings for launching events in Michotte’s setting and launching collisions in a photorealistically computer-rendered setting. We presented launching events with differing temporal gaps, the same launching processes with photorealistic billiard balls, as well as photorealistic billiard balls with realistic motion dynamics, that is, an initial rebound of the first ball after collision and a short sliding phase of the second ball due to momentum and friction. We found that providing the normal launching stimulus with realistic visuals led to *lower* causal ratings, but realistic visuals together with realistic motion dynamics evoked higher ratings. Two-dimensional versus three-dimensional presentation, on the other hand, did not affect phenomenal causality. We discuss our results in terms of intuitive physics as well as cue conflict.

As humans we are surrounded by interacting objects in everyday life, for example, while watching children play, during orderly to catastrophic interactions in the kitchen, while on the street during rush hour or while playing a game of billiard. Our mind constantly and typically unconsciously assigns causes to events in our surroundings. This gives us a unique ability to solve complex tasks (Pearl & Mackenzie, 2018). One of the prototypical and simplest cause–effect pairs is a collision between two objects. Displays of these events, so-called launching stimuli, are regarded as the canonical demonstration of causal perception (Wagemans et al., 2006). Albert Michotte (1963) was perhaps the first to systematically experiment with stimuli capable of evoking a feeling of causality. One of the displays he used became known as launching displays: two shapes (squares, now more commonly disks) starting some distance apart, one moving toward the other, at the point of contact the first disk stops and the second one continues its motion. He manipulated the properties of these displays with great experimental finesse and established many details about perceived causality. In his Experiment No. 29, he introduced a manipulation that has often been used in psychophysical research and thus earned its own name, *launching with temporal gap*. The sequence is the same, but between the stop of the first object and the start of the second there is a pause of a certain length where nothing moves: the temporal gap. The temporal gap is not natural, interfering with the causal continuation of the launching event; with longer temporal gaps, human observers experience a disruption of their causal impression.

The launching display has since been used to study the underlying mechanisms of causal perception in great detail (Boyle, 1960; Choi & Scholl, 2006; Guski & Troje, 2003; Leslie, 1982; Rips, 2011; Sanborn et al., 2013; Schlottmann & Anderson, 1993; Scholl & Tremoulet, 2000). By fulfilling the conditions necessary to evoke a causal impression as minimally as possible, launching makes it possible to precisely study the temporal dynamics of causal perception. In an influential study, Guski and Troje (2003) investigated the interaction between temporal gaps and additional auditory or visual markers in close temporal vicinity to the launching event. They found an increase in perceived causality if an auditory marker is added to the visual collision as well as a greater tolerance for temporal gaps if an auditory clack was provided. Beyond causal perception, scientists have also used this type of stimulus to study the perception of physical properties (Gerstenberg et al., 2012; Hubbard & Ruppel, 2017; Moors et al., 2017; Sanborn et al., 2013).

The availability of powerful rendering engines from computer graphics has created even more possibilities for manipulating the launching display. It also led to an increase in studies using realistic renderings of objects in launching. For example, M. E. Young et al. (2005) studied the perception of spatial and temporal contiguity in launching events. Wolff et al. (2014) used photorealistic and physically realistic-rendered stimuli to investigate the relationship between causal perceptions and the feeling of forces. Wang et al. (2018) used the same parameters and instructions of the Guski and Troje’s (2003) paper discussed earlier and replicated it in a virtual 3D environment. The quality of the Virtual Reality (VR)-rendered images in Wang et al. was, however, somewhat poor—clearly not photorealistic—and the background changed between two-dimensional (2D) and three-dimensional (3D) conditions. Thus, it is difficult to derive a definite conclusion regarding visual physical realism and 2D versus 3D influences on the launching event from their study. Recently, Bechlivanidis et al. (2019) investigated the relationship between causal impressions and different visual features. They used recorded movies and compared them with simplistic animations. They found “that impressions of causation depend predominantly on the core features and not the peripheral features of event sequences.” However, they noted that it would be important to better match properties in the animated conditions to the properties in the real-world conditions they used—exactly what we do in our study.

The purpose of this study is to study the relationship between Michotte launching events and physical realism in terms of both visual surface features (rendering) and motion dynamics (friction and momentum). Traditional experiments of causal perception, however, usually did not use displays of physical objects either because the necessary computer graphics were not yet available or because it was deemed important to separate the causal impression from physical reality (Sanborn et al., 2013; Schlottmann & Anderson, 1993). We, however, see no fundamental reason why collisions of physical objects should not be studied in the same framework as the collision of abstract, featureless disks. Quite to the contrary, we think the comparison between the two might shed light on the mechanism of phenomenal causality.

Our view is supported, we believe, by recent reports finding evidence for early perceptual—*peripheral features* —causal inference: Rolfs et al. (2013) found that humans observers show adaptation effects and negative aftereffects to launching events (but see Gallagher & Arnold, 2019, for a critique of the Rolfs et al.’s study). Furthermore, Kominsky and Scholl (2016) extended this line of work and showed that adaption to triggering displays leads to adaption of launching displays but not reverse. The human visual system seems to differentiate between categories of causal impressions (Kominsky & Scholl, 2018). Because of the retinotopic specificity of the adaptation effects, these two studies provide evidence for early, sensory processing of causality—and we thus think that it is worth exploring launching events using early, sensory manipulations like surface features and details of motion dynamics.

We altered the strength of the causal percept using time gaps between the motion onset of the second object, that is, we used the *launching with temporal gap*-paradigm. The experimental parameters and instructions to our observers were those of Guski and Troje (2003) in order to, first, replicate their original findings with abstract featureless disks and, second, if successful, enable us to interpret deviations for more physically realistic stimuli more convincingly. In addition, we not only photorealistically rendered the stimuli and used physically accurate motion taking momentum and friction into account but also displayed the stimuli with and without stereo cues (2D vs. 3D).

Finally, we are interested in the perceived difference between causal perception on an individual subject level. Thus, we used a *small N-Design*. A small N-Design means that we used only few participants, but that we recorded a large number of trials per participant. It has been shown recently that this design is often able to yield more stable results than traditional psychology approaches which use only a small number of trials but more subjects (Baker et al., 2019; P. L. Smith & Little, 2018).^1^ In a small N-Design, every subject is equivalent to a replication of the whole experiment because we attempt to show the effect in every single subject.

## Methods

### Subjects

Nine human subjects (five women and four men; aged 20 to 36 years; *M*: 25.4, standard deviation: 4.7) served as observers in our main study. Participants received monetary compensation. Written consent was collected before the experiment started. All experiments were conducted in accordance with the Declaration of Helsinki (World Medical Association Declaration of Helsinki, Version 2008). All participants reported normal or corrected-to-normal vision and passed a stereo test (OS-149; Western Optical Ophthalmic Instruments, Washington, United States).

### Apparatus

Stimuli were presented on a 24″, 120 Hz VIEWPixx/3D LCD monitor (VPixx Technologies, Saint-Bruno, Canada) with a resolution of 1,920 × 1,080 pixels. The screen was always viewed through 3DPixx LCD shutter glasses, thus effectively reducing the frame rate to 60 Hz for each eye—this was true for both the stereoscopic and nonstereoscopic stimuli in order to keep the viewing conditions constant (luminance, contrast, and color tint). Subjects reported their answers with a ResponsePixx button box. Observers kept a fixed distance of 70 cm to the display by the use of a chin rest. The experiment was programmed with the Psychophysics Toolbox (Kleiner et al., 2007; Version 3.0.15) in MATLAB (R2017b, The MathWorks, Inc., Natick, Massachusetts, United States) and was run on a dedicated 12-core Intel i7 Xeon desktop computer with an AMD Radeon HD 7970 graphics card running Linux (Debian 9).

### Stimuli

**Figure 1.**
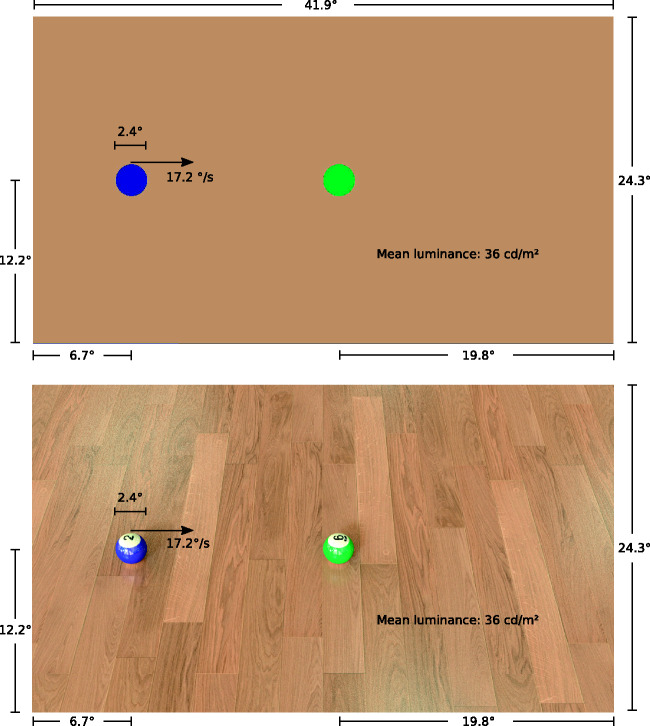
Visual Stimuli Used in the Experiment. The upper panel shows a crop from the launching condition which was originally proposed by Michotte. The lower panel shows the photorealistic-rendered stimulus. Both stimuli have identical spatial dimensions. All size measurements are in degree of visual angle for an observer viewing at a distance of 70 cm. The mean luminance of both displays was 36 cd/m^2^.

Stimuli were either classical uniformly colored Michotte launching disks or photorealistic-rendered versions of billiard balls. The blue disk or ball traveled from left to right on a computer screen and touched the green disk/ball. A temporal delay between 0 and 400 milliseconds was introduced before the green ball started to move. Adhering mostly to the experimental protocol from Guski and Troje (2003), we presented delays from 0 to 400 milliseconds in steps of 50 milliseconds, only leaving out 350 milliseconds as a prestudy had shown that ratings were almost the same for 350 and 400 milliseconds. Stimulus duration without gap was 1.65 seconds. Both conditions had the same spatio-temporal properties, see Figure 1, except for the physical realism where the spatio-temporal properties were controlled by Blenders physics engine.

In all conditions, disks had a diameter of 2.4° of visual angle and always traveled at a speed of 17.2 deg/s. The left disk started with its center at 6.7° and in the middle of the screen’s *y*-axis. The right disk had its left edge aligned to the middle of the screen and was also in the middle of the screen’s *y*-axis. The left disk then moved to the right and stopped just as it made contact. After the frame in which both disks just touched, the right disk moved to the mirror position of the left ones starting position, at which point the response screen with the background color of the Michotte condition appeared. The viewing distance was 70 cm. Mean luminance of the background was 36 cd/m^2^. The color of the background in the Michotte condition was the mean color (RGB: [185, 150, 108]) from the wooden floor in the Rendered conditions. The colors of the balls were matched to the underlying mapping of the colors in Blender. The color of the blue ball was [24, 41, 131] and the green color [0, 145, 78] in RGB coordinates. The geometry of the rendered scenes follows realistically laws. The viewing distance of the cameras in Blender was set to the viewing distance of the monitor, both 70 cm. The convergence plane was also set to 70 cm.

Photorealistic images were created in the 3D rendering software Blender v 2.79 using the Cycles renderer. The rendering included the effect of lightness, shadows, etc. The colors of the two balls and the background in the Michotte condition were chosen as the mean of the color of the corresponding object in the rendered images. The camera was above the ground (49.5 cm) and was tilted 45° toward the ground. We chose this angle to simulate a more natural, somewhat elevated viewing position for the physically realistic conditions. This is also compatible with the abstract Michotte version since the elevation angle is not constrained for the featureless 2D disks on a uniform background. The photorealistic condition was divided into two versions.

The first photorealistic condition consisted of two rolling balls. The momentum for rolling was added manually. We already added momentum to the balls since subjects in the pilot study (K. M., S. A. B., and one naive observer) reported very unnatural impressions with rendered (sliding) but nonrolling balls.^2^

The second Rendered condition modeled a physically correct interaction between balls and the surface. In this condition, effects of friction between surface and balls as well as friction between the balls at contact were included through the physics engine of the Cycles renderer. However, the friction coefficients in Cycles do not have a direct physical meaning and are arbitrary. To obtain physically correctly rendered interactions between the balls and the surface, we studied high-speed recordings of real launching events and modeled our balls accordingly.^3^ Adding the physically correct interactions only affected the motion sequence after contact, that is, the balls behaved in the same way as before for the first half of the display. Only after the collision both balls started moving as dictated by physics, leading to a short sliding phase of the green ball and movement of the blue ball due to its momentum and the friction, see Videos A1 to A6 in the Online Supplemental Material.

Both the Rendered conditions were in addition also presented with stereo cues. In this condition, the left eye and the right eye were rendered differently trough two separate cameras in Blender. The cameras had an interocular distance of 6.5 cm. Because of their transmission of less than 100%, the Nvidia shutter glasses led to a luminance decrease. Participants wore the glasses thus also in the nonstereo displays to ensure the same effective luminance of all stimuli in all conditions.

To summarize the section on the stimuli used in our experiments: We presented launching events with temporal gaps, using eight possible delays (0, 50, 100, 150, 200, 250, 300, and 400 milliseconds). The stimuli where either disks (Michotte) realistically rendered billiard balls (Rendered) or realistically rendered and physically correct moving billiard balls (Physical). The last two conditions were also shown additionally in stereo.

### Procedure

Every possible condition—combination of stimulus type and delay—was presented 20 times, resulting in 800 trials for each participant in total. The order of presentation was randomized; thus, all possible events were seen intermixed by every subject, allowing them to be judged on a single internal scale. Prior to data collection, every participant was presented with each condition exactly once in order to familiarize subjects with the range or spectrum of events. More importantly, this procedure helped the observers to anchor their subjective ratings. This is very important indeed, and we return to this issue in the Discussion section. After each presentation in the main experiment, the participants had to rate the event using the same question as used in Guski and Troje (2003, p. 792): “How probable is it that the movement of the [green] object (disk or ball) is caused by a perceivable event immediately before?” The rating was reported on a scale from 1 to 9, with 1 meaning *not at all* and 9 *very probable*.

The possible answers were displayed after each trial with a marker initially pointing at 5. The marker could be moved by pressing buttons on the ResponsePixx controller, which also allowed for a confirmation of the current position. Subjects had 3 seconds after the trial to give their answer, if not confirmed until then the current marker position was taken to be their answer. In practice, this happened only very rarely, in less than 4% of all trials. After the answer was confirmed or 3 seconds passed, a blank screen in the background color of the Michotte condition appeared for 1 second, and then the next event was shown.

### Data Analysis

Basic data analysis and plotting were done with the seaborn-package (Version 0.9.0) in Python (Version 3.7.3). In addition, we fitted a Generalized Additive Mixed Model (GAMM) with the mgcv package in the statistical language R (R Core Team, 2019). For an introduction to GAMMs, see Wood (2017). In the easiest case, a Linear Additive Mixed Models extends the linear model:

(1) $$y=\text{β}x+\epsilon ,\;\epsilon ∼N(0,{\text{σ}}^{2}),$$

of variables *x*, *y*, and parameter β to

(2) $$y=\text{β}x+\text{γ}z+\epsilon ,\;\;\text{γ}∼N(0,\phi^{2}),\;\;\epsilon ∼N(0,{\text{ρ}}^{2}),$$

where γ contains random effects with zero expected value, and covariance ρ; *z* is the model variable for these random effects. One assumption of a standard linear model is that the residuals are independent. In a Linear Mixed Model, this assumption is relaxed. Consider in our study that we collected data from different subjects. Some subjects might have a bias toward higher or lower ratings. Thus, the residuals are not independent anymore. The variable *z* allows modeling this nonindependence within one subject. In our study, a mixed model offers the opportunity to model different influences on ratings in an additive effect, for example, effects of subjects or conditions. Separate models were run to find an appropriate model structure. Model comparison was done with the Akaike Information Criterion (Akaike, 1974). Our best fitting GAMM models the rating as a linear sum of an offset per condition, a separate smooth function over delays for every condition, and a random effect of subjects over delays. More technical details are presented in the Online Supplemental Material.

## Results

The purpose of this work is to study the relationship between visual and physical cues in launching displays. To this end, we first investigated the relationship between stereo and nonstereo cues. There were no systematic trends in the effect of stereo cues on perceived causality as shown in Figures S.1a and S.1b in the Online Supplemental Material. Thus, in the following, we show the corresponding ratings from stereo and nonstereo conditions pooled within observers. This doubled the effective sample size for the Rendered and Physical conditions to 40 trials per observer and delay.

**Figure 2.**
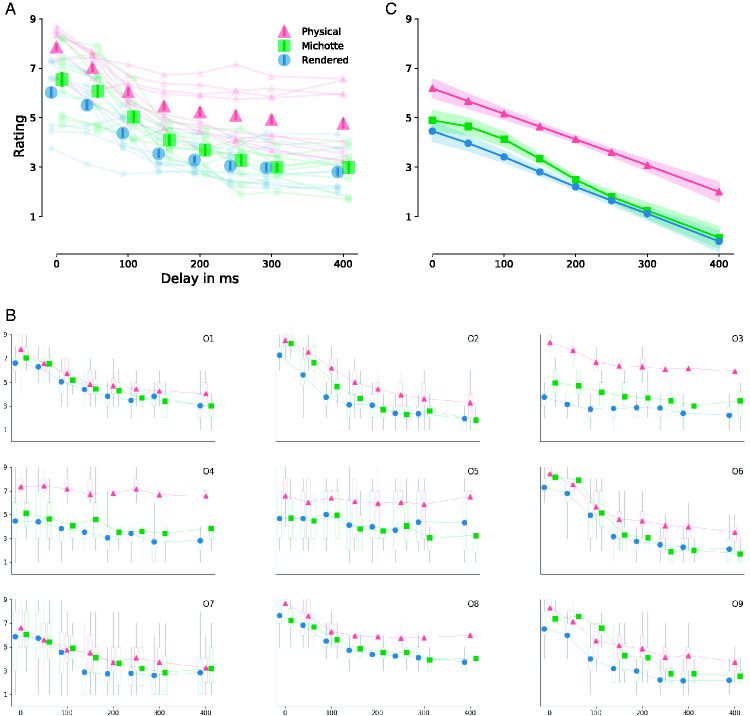
Mean Causal Ratings for the Three Conditions. A: Mean result of the different conditions from pooled data across all subjects. B: Mean causal ratings for all individual observers. The error bars indicate bootstrapped 95% confidence intervals; outliers not shown for clarity. The confidence intervals for the Michotte condition are larger because they come from fewer data points than the Rendered and Physical conditions, which have been pooled with their stereo counterpart. C: Results of the GAMM with removed random subject effects. Shaded area indicates 95% confidence interval.

The mean ratings for the Michotte, Rendered, and Physical conditions for each delay are shown in Figure 2A. What can be seen is the typical decline of causality ratings with increasing delay, and a tendency for an asymptotic *lowest causality* rating for the longest delays. This general trend can also be found in individuals ratings (Figure 2B). While the precise shape of the curves varies between individuals, typically the Rendered and Physical curves are very similar, simply translated by a value of 1.8. Some subjects also show the trend seen in the pooled data that for long delays the Michotte displays receive ratings close to the Rendered condition.

The ranking of conditions is very clear, for a certain delay the physically realistic display receives the highest causal ratings, followed by the Michotte display, and lastly the realistically rendered display. This ordering can also be found in most individuals (sometimes, there is no clear ordering between the conditions, in particular for the Michotte and the Rendered condition, but the order never substantially deviates from the mentioned trend). The effects are substantial: The Rendered condition at 0 milliseconds delay, for example, appears only as causal as the Physical condition at 100 milliseconds; at 100 milliseconds delay, the Rendered condition matches the Physical condition at 300 milliseconds in its causal appearance. We return to this in the Discussion section and Figure 4.

Results from our fitted GAMM are shown in Table 1. The GAMM explains 53% of the variance. The condition intercept terms are significant, meaning that each condition yields a significant offset. In addition, each conditions smooth term is highly significant, thus each condition follows a nonlinear path/trajectory. Finally, the random smooth terms associated with the subject effect are highly significant. Figure 2C plots results from the GAMM with removed random subject effects. This plot shows the effect of condition on ratings across delays without the effect of the individual subjects. It clearly shows that the Rendered condition is seen as least causal at all delays, the Physical condition as most causal; the abstract Michotte condition is in-between.

**Table 1.** Summary of the Best Fitting Model.

A. Parametric coefficients	Estimate	Standard error	*t*	*p*
(Intercept)	2.84044	0.08464	33.560	<.0001
ConditionRendered	−0.38645	0.04850	−7.968	<.0001
ConditionPhysical	1.46737	0.04850	30.256	<.0001
B. Smooth terms	Estimate *df*	Ref.*df*	*F*	*p*
s(Subject, Delay)	34.224	37.0000	68.08	<.0001
s(Delay): ConditionMichotte	3.917	3.917	66.35	<.0001
s(Delay): ConditionRendered	2.970	2.970	81.98	<.0001
s(Delay): ConditionPhysical	1.160	1.160	135.98	<.0001

*Note.* Small *p* values indicate significant effects.

**Figure 3.**
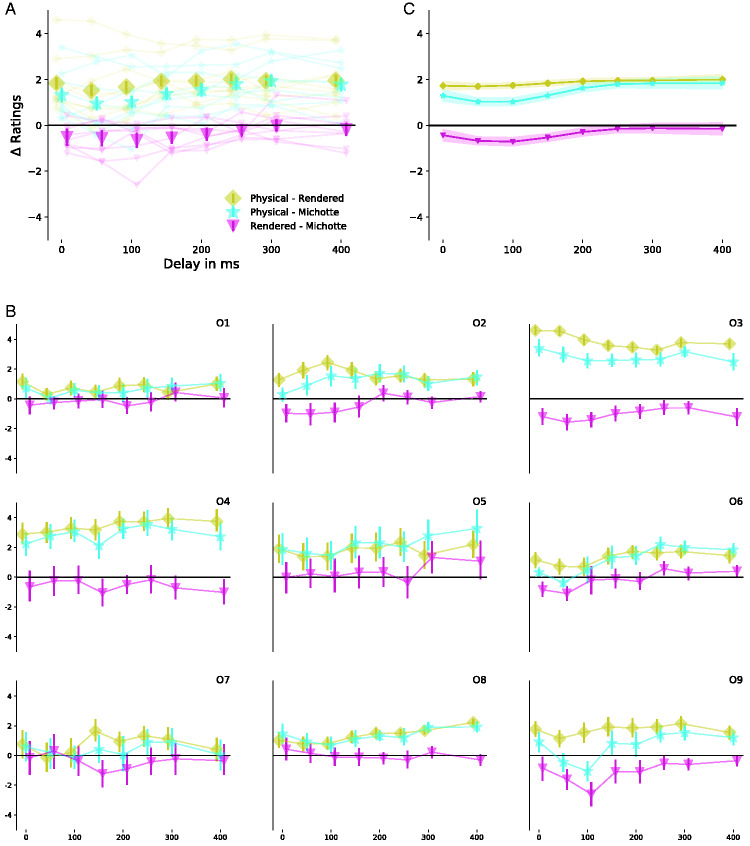
Difference for Ratings Between All Three Conditions. A: Results of the different condition from pooled data across all subjects. The error bars indicate bootstrapped 95% confidence intervals. B: Mean difference for individual observers. C: Results of the GAMM. Shaded area indicates bootstrapped 95% confidence intervals.

We further examined the effect of individual subjects on ratings. Therefore, we looked at the difference of ratings between conditions. Figure 3A shows the mean differences pooled across all observers, whereas Figure 3B shows the results for individual observers. The difference between Rendered and Michotte and between Physical and Michotte shows a remarkably similar trend (Figure 3A). It seems that they have the same functional form with a shifted offset. The underlying assumption of our GAMM states that subjects have an additive effect regardless of condition. Therefore, we should expect that all subjects show the same result for differences between conditions. Figure 3C shows the result of the GAMM analysis. Figure 3B shows that all subjects behave indeed qualitatively similar. This is a post hoc corroboration for the use of GAMMs to analyze our data.

## Discussion and Conclusion

We explored the effects of substituting realistic visuals and physics into Michotte launching displays. All three conditions (Michotte, Rendered, and Physical) were intermixed, and a small number of subjects evaluated them on a common scale with a large number of repetitions.^4^ Clearly, rating scales are not the first choice if one wants a stable, reliable, and precise means to quantify human perception, and in most situations, performance-based methods are probably preferable (Wichmann & Jäkel, 2018). This criticism was, incidentally, already raised against Michotte himself (Joynson, 1971). Some authors therefore used nonrating methods in creative ways in their investigations of phenomenal causality (Kominsky et al., 2017; Moors et al., 2017; Rolfs et al., 2013; Scholl & Nakayama, 2004). In our study, our first aim was, however, to measure the *subjective experience* of how causal the stimuli looked: Thus, we had our observers rate their causal impression. Second, we aimed at being as close as possible to the original Michotte experiments and be able to compare our results with existing experiments using the very same experimental parameters (Guski & Troje, 2003; Wang et al., 2018, see Supplemental Material *Comparison to previous data*). Finally, we took great care to minimize factors known to make rating scales unreliable: We showed the range of stimuli to the observers prior to the experiment to help them as much as possible to anchor their scales. All conditions were interleaved to avoid serial position effects or re-anchoring to contaminate the ratings between conditions.^5^ Finally, as mentioned earlier, we chose a small N-Design with many repetitions to be able to show the effect on the level of individual observers (see Figures 2 and 3). Together with the clean results from our GAMM analysis, we have confidence in our results despite them being of the rating scale type.

**Figure 4.**
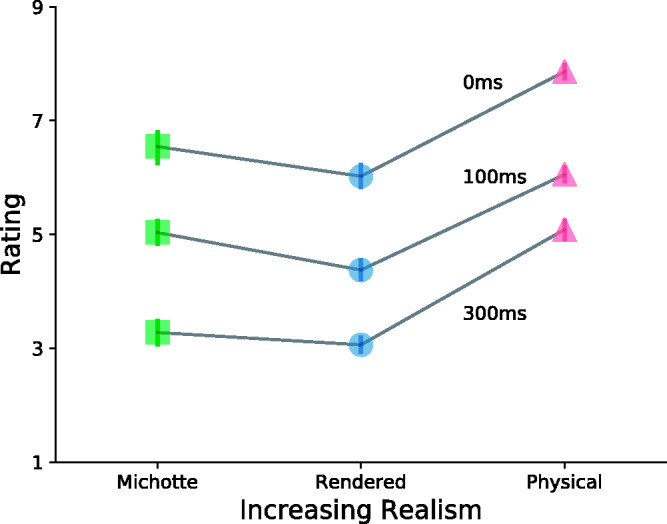
Effect of Increasing Realism on the Mean Rating Over All Subjects for Three Chosen Delay Lengths. Adding realistic visuals to typical Michotte Launching lead to a dip in causal ratings. The strength of the causal percept is only recovered, and in fact exceeded, when realistic visuals are paired with realistic physics, no matter the delay length. Thus, the process of going from abstract to realistic exhibits a kind of Uncanny Valley effect. Error bars indicate bootstrapped 95% confidence intervals.

Figure 4 shows causal ratings across all conditions for three different delays: 0, 100 and 300 milliseconds. Realism increases from left to right. We can observe that simply turning the disks of classical launching into photorealistically rendered billiard balls in a realistic environment *weakens* phenomenal causality. The change toward more visual realism seems to bring with it a demand for more physical realism of the motion dynamics. If this demand is met in our Physical condition, however, the causality ratings even trump those of the Michotte display (which, if not compared with realistic physics, is usually perfectly capable of evoking the highest ratings). This effect has a semblance to the uncanny valley effect from robotics (Mori et al., 2012): If realism is almost, but not quite attained, a strong feeling of unnaturalness—and often unease with humanoid robots—is evoked.^6^

Our results can also be interpreted in light of the intuitive physics debate; proponents believe that humans possess a rather detailed but largely unconscious knowledge of (approximate) physics—typically approximately correct to enable action in the world rather than following the exact laws of physics (Battaglia et al., 2013; Kubricht et al., 2017; K. A. Smith et al., 2013; Ullman et al., 2017). The pattern of movement in the Physical condition is closer to everyday observed collisions and would thus evoke a stronger feeling of causality. Another possible explanation is that there is more evidence for a singular collision event in the Physical display. The backlash of the first ball hints at an actual collision, and the initial sliding instead of rolling of the second ball might be taken as an indication that it was initiated by an outside force (and not say, started by its own volition). The co-occurrence of these elements might serve as evidence for an event, an exchange of kinetic energy, thereby strengthening the causal percept. In addition, it is well known that observers underestimate the physical effect of the second ball on the first ball (White, 2006). Within this context, it may be helpful to run a new experiment similar to that of Vicovaro (2018), who analyzed launching events but with manipulated velocity of the second disk. Smith et al. (2013) showed that observers can use their intuitive physics to predict where a ball released from a pendulum lands, even though they cannot correctly draw the trajectory. We ourselves initially perceived the physically correct display as somewhat unrealistic, even though we also perceived the event as strongly causal—none of the authors initially felt that something was missing from the event in the Rendered condition; but compared with the Physical condition, the Rendered condition evoked a much less vivid impression of phenomenal causality.^7^ An in-depth extension of our study exploring the connection between realism of the motion dynamics and causality in our specific case may be worthwhile.

An alternative—and not necessarily mutually exclusive—interpretation of our results is not in terms of the Uncanny Valley effect but instead as one of different levels of cue conflict (Landy et al., 1995; M. J. Young et al., 1993). Experimentally presenting conflicting cues, for example, sounds of an event coming from one direction, while visual information indicates the other is a popular manipulation (McGurk & MacDonald, 1976; Sekuler et al., 1997; Shams et al., 2000). In this view, the Michotte condition is consistently unrealistic, the Physical condition is consistently realistic, but the Rendered condition is inconsistent in its relationship to reality and thus one of maximal cue conflict (Hoffman et al., 2008; Rosas & Wichmann, 2011). The almost parallel dependence of Physical and Rendered ratings as a function of the delays—most visible in the GAMM analysis, see Figure 2C—may be evidence that they are evaluated by the same mechanism, but the cue conflict in the Rendered condition subtracts a constant offset from the strength of the experienced causal percept.

However, it is important to note that not all visual cues influence causal ratings equally. We found that while only adding realistic visuals to launching displays decreases causal ratings, additionally adding realistic motion dynamics yields higher causal ratings than the Michotte baseline—increasing realism by adding stereo cues, on the other hand, did not affect causality ratings at all, see Figure S.1. Furthermore, even in the Rendered condition, we made the billiard balls roll, since subjects in a pilot study (K. M., S. A. B., and one naive observer) reported very unnatural and clearly noncausal impressions with rendered but nonrolling (sliding) balls. This, again, stresses the importance of ensuring “equal levels” of realism—avoiding strong cue conflicts—for both visuals and motion dynamics in the Michotte launching paradigm.

Thus, we would like to modify the conclusions made by Bechlivanidis et al. (2019). Yes, causal impressions in our experiment depend on so-called core-features, for example, the delay and the fact that two objects interact. But, on the other hand, we also found that altering *peripheral features* could strongly influence the causal percept. Thus, we conclude that also early or peripheral sensory cues are important for the perception of causality. The precise mechanism underlying this effect is still unknown, and there are many possible directions to explore at this point. It might also be interesting to try and create a stimulus with Michotte visuals, but realistic motion dynamics, for example, by just drawing the Physical condition with Michotte-like 2D disks (the otherwise uniform disks would need to have a single surface marker off-center to allow their rotation and sliding to be seen).

We think a better understanding of the relationship between the percept evoked by Michotte launching and the one resulting from realistic collisions is a valuable pursuit—ideally we would like to know all the stimulus features or cues the human visual system uses when perceiving causality. A highly relevant study speaking to this issue is that by Kominsky et al. (2017). Kominsky et al. showed that human observers are highly sensitive to Newtonian constraints on the *velocity* of the disk set in motion by the first disk using abstract, Michotte-style stimuli. This would be especially interesting in light of the uncanny valley discussion earlier. Like our results, this suggests that the visual system has detailed, internalized knowledge about the physics of real-world collisions. Whether or not, or to which degree, both abstract Michotte launching and realistic collisions engage the same sensory and cognitive processes might aid in the question of where in the perceptual hierarchy the perception of causality is situated.

## Supplementary Material

Supplementary material

Supplementary material

Supplementary material

Supplementary material

Supplementary material

Supplementary material

Supplementary material

## Notes

1 The choice depends on whether one wants to estimate populations parameters or is interested in the individual mechanism for every participant. If interested in the latter, as we are, small N-Designs with many trials per participant are preferable (P. L. Smith & Little, 2018).

2 We will return to this issue in the discussion when discussing sensory realism and cue conflict.

3 We used the following YouTube video as reference for modeling the effects of friction in Blender: Realexperiment—Zentraler Stoß zweier gleichschwerer Billardkugeln. Retrieved from https://www.youtube.com/watch?v=ww1NC74HtL8 User: nighthawk1158, July 27, 2013.

4 The trends we found in our Michotte condition are similar to those reported by other researchers albeit somewhat cleaner and more consistent due to our small N-Design, see Figure S.2 in the Online Supplemental Material for a comparison of our data to those of Guski and Troje (2003) and Wang et al. (2018).

5 We would like to point out that the range of presented stimuli—and when and how observers see the full range—very likely has an enormous influence on the reported causality ratings: anchoring. In the original Michotte Experiment No. 29, Michotte only used gaps between 0 and 180 milliseconds and found that already for gaps longer than 150 milliseconds, no causal impressions were reported. In our setting with gaps of up to 400 milliseconds, we obtained intermediate causal ratings even at 400 milliseconds—consistent with the results of Guski and Troje (2003) and Wang et al. (2018), see Figure S.2 in the supplemental material. M. E. Young et al. (2005) used stimuli with gaps up to 2,000 milliseconds (!) and, in addition, spatial gaps of up to 4 cm. Given this large range of variation observers in this study still reported moderate causal impressions for the 2,000 milliseconds gap without spatial gap—presumably because, compared with the condition with a 4 cm spatial gap, the no spatial gap condition felt “a bit more causal” than the spatial gap condition.

6 One of our reviewers offered an alternative interpretation of our results: Perhaps adding surface feature information, that is, visual realism, to the Michotte condition distracts the visual system. This is only compensated when the correct motion dynamics are also added. Evidence for this line of argument may come from a finding from infant research: Infants at 6 months of age showed sensitivity to causal relationships in Michotte displays but with visual realistic stimuli this response starts at only 10 months (Cohen & Amsel, 1998; Oakes & Cohen, 1990).

7 Although it is important to note that the movement of the second ball, especially its spin, depends on the movement and spin of the first ball (backspin, forward spin, etc.)—however, we believe that only connoisseurs of billiard will know all the intricacies and exact dependencies. We believe what matters is that there is some nonzero spin, momentum, and friction consistent with Newtonian physics.
