# Supplementary material for: Phenomenal Causality and Sensory Realism
Source: Iperception. 2020 Jun 1;11(3):2041669520927038. doi: 10.1177/2041669520927038 (PMC7268924; doi:10.1177/2041669520927038)
Supplement: Supplementary material [file Supplementary_fig_tables.pdf]

Supplemental material

Stereo and non-stereo condition

a

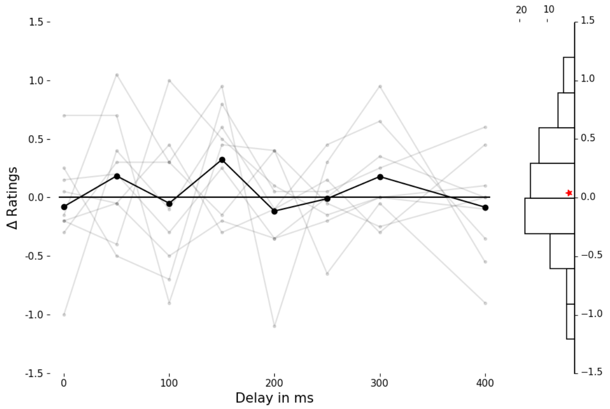

b

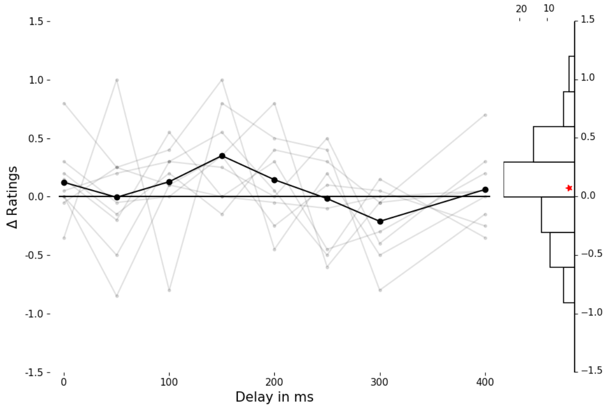

Figure S.1. Comparison of stereo to the non-stereo condition of Rendered (a) or Physical (b) data. The bold black line shows the mean difference between the stereo and non-stereo condition across all subjects. The x-axis shows the Delay in ms and the y-axis the differences between conditions. Light lines show all eight individual subject differences. The Histogram to the right shows the distribution of individual differences. A Shapiro-Wilk normality tests show that both distributions are not significantly ($p < 0.01$) different from a normal distribution (a: W=0.987, p = 0.66, b: W=0.981, p = 0.36). Additionally, a t-test shows that both means are not significantly different from zero (a: μ= 0.04, t = 0.78, p = 0.44, b: μ=0.07 , t=1.53, p = 0.13).

Comparison to previous data

a

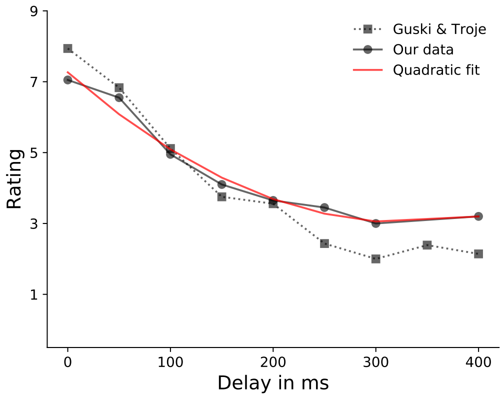

b

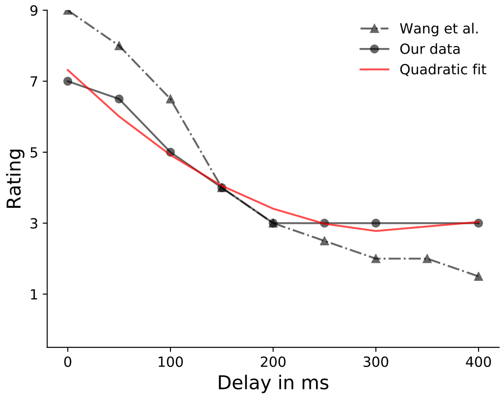

Figure S.2. Comparison to previous data from Guski and Troje (2003) and Wang et al. (2018). Data for both Figures were extracted from the original papers; because they used different plotting conventions, they cannot be shown in one graph. (a) Guski and Troje calculated the mean of individuals and then plotted the median of these means. They showed that their data have a quadratic trend ($r^2 = 0.99$). Our data for the Michotte condition also show a quadratic trend (quadratic: r^2: 0.98 BIC: -18.62, linear: r^2: 0.81, BIC: -3.29, cubic: r^2: 0.98, BIC: -16.63). The quadratic fit to our data is shown in red. (b) Wang et al. used median ratings. Wang et al. fitted linear, quadratic, cubic and logistic regression lines. Cubic regression lines fitted best. In our case on the other hand the quadratic trend proved to be superior here, too, as found by Guski and Troje (2003) (quadratic: r^2: 0.97 BIC: -14.92, linear: r^2: 0.78, BIC: -0.93, cubic: r^2: 0.97, BIC: -13.22). The quadratic fit to our data is shown in red.

Technical details of the GAMM analysis

We provide the full summary of our GAMM in R below.

```
Family: gaussian
Link function: identity

Formula:
Rating ~ Condition + s(Subject, Delay, bs = "fs", k = nKnots) +
    s(Delay, by = Condition, k = nKnots, bs = "ds")

Parametric coefficients:
             Estimate Std. Error t value Pr(>|t|)
(Intercept)   2.84044    0.08464  33.560  < 2e-16 ***
ConditionRR  -0.38645    0.04850  -7.968 1.86e-15 ***
ConditionRRP  1.46737    0.04850  30.256  < 2e-16 ***
---
Signif. codes:  0 *** 0.001 ** 0.01 * 0.05 . 0.1   1

Approximate significance of smooth terms:
                          edf Ref.df      F p-value
s(Subject,Delay)       34.224 37.000  68.08  <2e-16 ***
s(Delay):ConditionAbst  3.829  3.917  66.35  <2e-16 ***
s(Delay):ConditionRR    2.583  2.970  81.98  <2e-16 ***
s(Delay):ConditionRRP   1.092  1.160 135.98  <2e-16 ***
---
Signif. codes:  0 *** 0.001 ** 0.01 * 0.05 . 0.1   1

Rank: 49/60
R-sq.(adj) =  0.527   Deviance explained =   53%
GCV = 2.2723  Scale est. = 2.2583    n = 7200
```

We used a Gaussian GAMM, meaning that the conditional distribution of the response ratings follows a Gaussian distribution. The link function is the identity, strictly speaking, we are not using a *Generalized* Additive Mixed Model but only a Linear Additive Mixed Model. The formulas above show the details of the final model. Ratings are modelled as the sum of an intercept based on the condition, smooth terms over the delays for each condition and a random smooth term for every individual subject. All other tested models, as well as their AIC scores, are presented in table S.1. We used Duchon splines (bs =”ds”) for conditions’ smooth functions. Standard smooth functions are thin plate regression splines. We set the number of basis functions to 5 (k = ”5”). This is, in general, a quite small number for a GAMM analysis but we used only 8 different delay positions. Therefore k must not exceed 8. The subject dependent smooths were modeled with factor smooth interactions (bs = ”fs”). Factor smooth interactions model for each subject a quasi-random smooth function. We checked and confirmed our choices with the *gam.check* and autocorrelation function.

Table S.1. Model comparison with the AIC for different GAMMs. Best fitting model is highlighted in bold and used for our analysis.

GAM-model	AIC
Rating = Condition + s(Delay)	27782
Rating = Condition + s(Delay,Condition)	27754
Rating = Condition + s(Delay,Condition) + s(Subject)	27283
**Rating = Condition + s(Delay,Condition) + s(Subject, Delay)**	**26344**
Rating = Condition + s(Delay,Condition,Delay)	26852
